# Activation of blood coagulation in cancer: implications for tumour progression

**DOI:** 10.1042/BSR20130057

**Published:** 2013-09-04

**Authors:** Luize G. Lima, Robson Q. Monteiro

**Affiliations:** *Instituto de Bioquímica Médica, Universidade Federal do Rio de Janeiro, Rio de Janeiro, Brazil; †Centro de Transplante de Medula Óssea, Instituto Nacional de Câncer, Rio de Janeiro, Brazil

**Keywords:** blood coagulation, metastasis, protease-activated receptor, tissue factor, Trousseau’s syndrome, tumour growth, FIX, factor IX, FVIIa, factor VIIa, FVIII, factor VIII, FX, factor X, MMP, matrix metalloprotease, MV, microvesicle, NET, neutrophil extracellular trap, NK, natural killer, PAR, protease-activated receptor, PS, phosphatidylserine, TF, tissue factor, VEGF, vascular endothelial growth factor

## Abstract

Several studies have suggested a role for blood coagulation proteins in tumour progression. Herein, we discuss (1) the activation of the blood clotting cascade in the tumour microenvironment and its impact on primary tumour growth; (2) the intravascular activation of blood coagulation and its impact on tumour metastasis and cancer-associated thrombosis; and (3) antitumour therapies that target blood-coagulation-associated proteins. Expression levels of the clotting initiator protein TF (tissue factor) have been correlated with tumour cell aggressiveness. Simultaneous TF expression and PS (phosphatidylserine) exposure by tumour cells promote the extravascular activation of blood coagulation. The generation of blood coagulation enzymes in the tumour microenvironment may trigger the activation of PARs (protease-activated receptors). In particular, PAR1 and PAR2 have been associated with many aspects of tumour biology. The procoagulant activity of circulating tumour cells favours metastasis, whereas the release of TF-bearing MVs (microvesicles) into the circulation has been correlated with cancer-associated thrombosis. Given the role of coagulation proteins in tumour progression, it has been proposed that they could be targets for the development of new antitumour therapies.

## INTRODUCTION

In the 1860s, the French physician Armand Trousseau reported the occurrence of ‘mysterious’ thrombotic disorders in cancer patients and concluded that spontaneous blood coagulation events are frequent in these individuals because of a ‘special crisis in their blood’ [1]. Later, his name was used to designate the manifestation of thrombophlebitis in patients with malignant neoplasias-Trousseau's Syndrome. Currently, this designation commonly comprises all cases where unexplained thrombotic events precede the diagnosis of an occult malignant tumour or appear concurrently with the tumour.

Since the publication of ‘Clinique Médicale de l’Hôtel-Dieu de Paris’ by Trousseau in 1865 [1], an important link between malignancy and hypercoagulable states has been established [2,3]. In fact, the occurrence of cancer is usually associated with various clinical thrombotic syndromes, including local and systemic venous and arterial thrombosis [4]. Additionally, thrombosis is often diagnosed as the first clinical manifestation of a tumour and the second leading cause of death of patients with cancer [3,5,6]. It is noteworthy that abnormalities in *in vitro* coagulation tests are found in more than 90% of patients with cancer, irrespective of their thrombotic status [7].

Various authors have demonstrated a significant correlation between the incidence of thromboembolic events and a worse prognosis of neoplastic disease, supporting the idea that the activation of the blood coagulation system contributes to tumour aggressiveness and vice versa. Sorensen et al. [8] noted that the first-year survival rate of patients who are diagnosed with both cancer and venous thromboembolism was 12%, in contrast with 36% observed in cancer patients without a diagnosis of thromboembolic events. Patients with thrombosis-associated malignancies were also reported to exhibit a higher mortality in the first 6 months of a thrombotic event than those individuals presenting with cancer without thrombosis or thrombosis without cancer [9]. It is important to note that the lower survival rate observed in cancer patients displaying a thrombophilic profile is not necessarily related to the thrombotic event itself but probably to tumours with a more aggressive behaviour. Sallah et al. [10], for example, demonstrated that the occurrence of disseminated intravascular coagulation (a consumptive coagulopathy) in patients with solid tumours had a negative effect on the survival of those individuals, regardless of the manifestation of thrombosis. These results suggest that the haemostatic system may play an important role in cancer pathogenesis. Indeed, a large body of evidence has indicated that cellular and circulating haemostatic factors have an active role in the fundamental aspects of tumour biology, such as the angiogenesis, metastasis and modulation of innate immune responses [11,12]. The experimental strategies applied include pharmacological modulation of the function of platelets and various constituents of the blood coagulation cascade, as well as the use of genetically modified animals exhibiting altered expression or activity of those components.

### Molecular and cellular bases of haemostatic activation in cancer

Corroborating clinical data indicate a close association between tumour progression and the development of a thrombophilic profile. Diverse cellular and molecular evidence linking cancer with a hypercoagulable state has been described. Histopathological analyses demonstrate the presence of fibrin deposition and platelet aggregates in and around different tumours, indicating local activation of coagulation [13]. In addition, haemostatic alterations analysed by the laboratory tests are found in 60–100% of patients with malignant neoplasias, including those without thrombotic manifestations [7]. These changes comprise different levels of blood coagulation abnormalities, such as shortened aPTT (activated partial thromboplastin time), elevated levels of circulating blood coagulation proteins [i.e., fibrinogen, FV (factor V), FVIII (factor VIII), FIX (factor IX) and FX (factor X)], thrombocytosis and increased concentrations of fibrin/fibrinogen degradation products, among others [14].

These findings can be partly explained by the inflammatory response associated with neoplasia, by a change in protein metabolism and/or by venous stasis. However, various studies have demonstrated the importance of the participation of specific procoagulant properties of tumour cells, including the expression of TF (tissue factor), the central trigger of the coagulation cascade; the surface exposure of the phospholipid PS (phosphatidylserine), which provides a negatively charged surface required for the assembly of some catalytic active coagulation complexes; and the shedding of circulating procoagulant MVs (microvesicles). In the present review, we will discuss the different mechanisms of blood coagulation activation in cancer and their role in tumour progression.

## EXTRAVASCULAR ACTIVATION OF BLOOD COAGULATION IN CANCER

### Molecular mechanisms

The blood coagulation cascade is initiated upon the binding of FVIIa (activated factor VII), a plasma protein, to TF, a 47-kDa transmembrane protein that is constitutively expressed on the surface of subendothelial cells and some extravascular tissues [15]. This binding leads to the proteolytic activation of various coagulation zymogens, such as FX, FIX and prothrombin, resulting in the formation of a fibrin clot. Interestingly, TF expression is up-regulated on the surface of transformed cells, which has long been implicated in the *in vitro* procoagulant activity and cell aggressiveness of different tumour cell lines [16–18]. Moreover, TF was shown to be overexpressed in samples from patients with various neoplasias, including most carcinomas and other tumours such as melanoma [19].

TF overexpression in malignant tumour cells seems to be directly related to oncogenic events such as the presence of the mutant oncogenes K-*ras*, EGFRvIII (epidermal growth factor receptor variant III) and HER-2 (human epidermal growth factor receptor 2), as well as the loss of the tumour suppressor genes p53 and PTEN (phosphatase and tensin homologue) [20–22]. Furthermore, Zhang et al. [23] showed that the selective up-regulation of TF in highly invasive MDA-MB-231 human breast cancer cells, compared with that in less invasive MCF-7 cells, also appears to be regulated by miRNA-19 (microRNA-19).

**Figure 1:**
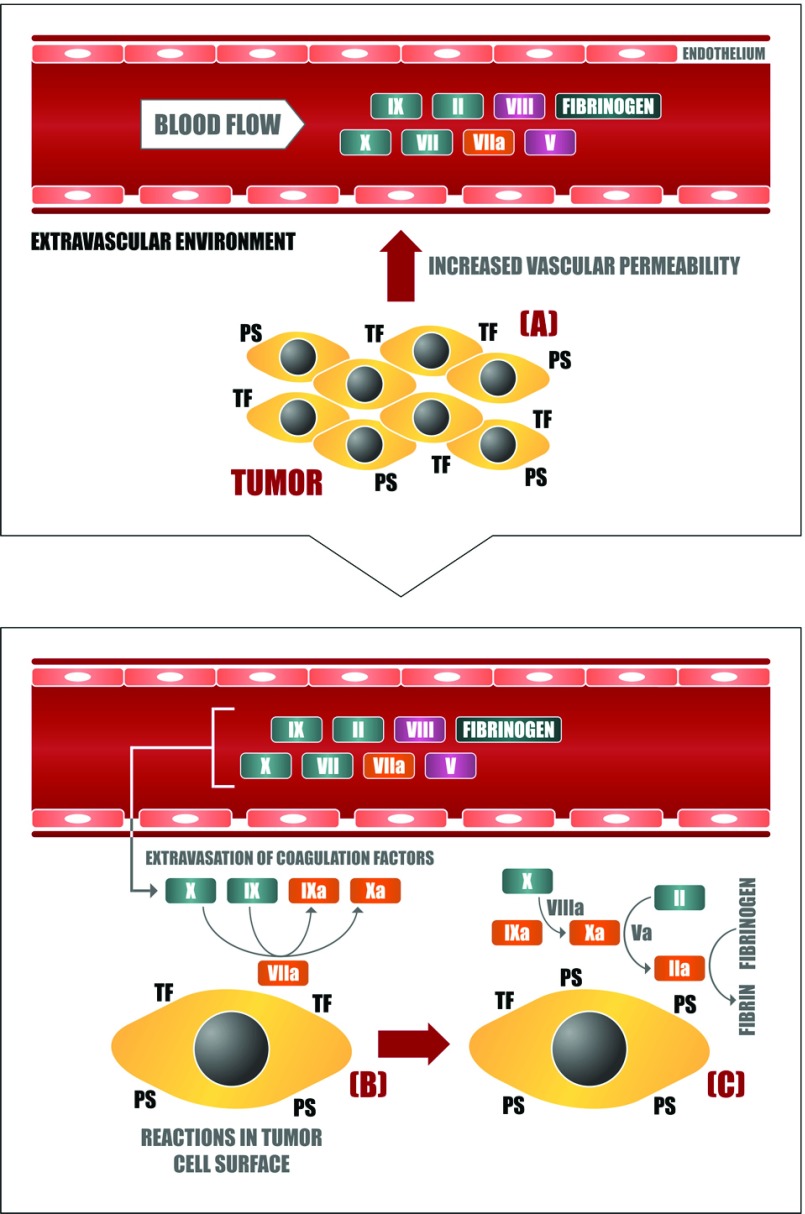
Extravascular activation of blood coagulation in cancer (**A**) Neoplastic cells stimulate vascular permeability in the tumour microenvironment through the generation of proangiogenic factors such as VEGF. In this way, blood coagulation proteins can leave the plasma circulation and reach the extravascular tumour microenvironment, getting in contact with the plasma membrane of tumour cells, which is rich in procoagulant molecules such as (**B**) TF and (**C**) PS. The assembly of different blood coagulation complexes thus culminates in the local generation of thrombin and fibrin.

Tumour cells exhibit higher levels of the phospholipid PS on their membrane outer surface (compared with normal cells) [24], supporting the assembly of blood coagulation complexes that depend on negatively charged membranes. Thus, by exposing TF and PS on their outer membrane, tumour cells can function as binding surfaces for different proteins of the coagulation cascade (i.e., factors VIIa, VIIIa, IXa, Xa and Va) [25,26] and promote the subsequent assembly of the prothrombinase and tenase complexes, leading to the generation of fibrin in the extravascular environment (Figure 1). TF expression by tumour cells allows for the formation of the extrinsic tenase complex; establishment of the intrinsic tenase and prothrombinase complexes on the surface of various tumour cell lines has also been described, mainly associated with PS exposure [27–31]. Remarkably, data obtained with cell lines are consistent with results from immuno-affinity ligand-binding studies that have demonstrated the presence of FXa (activated FX) and thrombin in human patient tissues [32,33].

### Impact of coagulation proteins on primary tumour growth

In cancer patients, TF expression in different neoplasias has been correlated with tumour grade, increased vascular density and worse prognosis [34,35]. Additionally, *in vitro* studies have shown that a strong correlation exists between TF expression and VEGF (vascular endothelial growth factor) production [36]. In fact, TF expression by different cells present in the tumour microenvironment (neoplastic or stromal ones) has been particularly related to tumour growth and metastasis-associated events. These include thrombin generation, and its subsequent interaction with multiple targets, as well as the stimulation of some surface receptors that are activated by many proteolytic enzymes, including blood coagulation serine proteases. These receptors, referred to as PARs (protease-activated receptors), are typical seven-transmembrane, G protein-linked receptors that are activated by a unique mechanism. Proteases cleave the amino terminus of PARs, allowing the internal ligand to autoactivate [37]. Of the four mammalian PARs, PAR1, PAR3 and PAR4 can be activated by thrombin, whereas PAR2 can be activated by coagulation proteases FVIIa and FXa but not thrombin [37–39]. These receptors are expressed in various tissues, where they are involved in a number of physiological and pathological phenomena [40].

PARs are usually overexpressed in various human cancer types, and many studies have shown that a strong correlation exists between their expression and aggressive behaviour of tumour cells [41–45]. The proteolytic activation of these receptors in tumour cells triggers complex signalling mechanisms that can stimulate migratory and/or invasive abilities and the production of chemotactic and proangiogenic factors, such as IL-8 (interleukin-8) and VEGF [46]. *In vitro* and *in vivo* studies have demonstrated, for example, that the intracellular signalling promoted by thrombin, through the proteolysis of PAR1 in tumour and endothelial cells, elicits a proangiogenic process that is associated with VEGF production and signalling, as well as the secretion of MMPs (matrix metalloproteases) [47–49].

Most of the pro-tumoural functions of TF concerning angiogenesis and primary growth have been correlated with the intracellular signalling triggered by TF binding to FVIIa and FVIIa/FXa-mediated proteolysis of PAR2 [50–56]. Remarkably, some of these effects seem to be independent of the procoagulant activity of the TF/FVIIa complex, because they are sustained even when the capacity of TF/FVIIa to promote FXa generation is completely blocked [57]. PAR2 activation has been correlated with the production of tumour-promoting molecules, primary tumour growth and the proangiogenic and invasive properties of cancer cells [58,59]. In this context, blockade of TF-mediated signalling through PAR2 using TF or PAR2 antibodies decreases primary tumour growth and reduces tumour angiogenesis in a human breast cancer model [57]. These observations are supported by studies employing a spontaneous murine breast cancer model in which a PAR2, but not PAR1, genetic deficiency delays tumour growth and angiogenesis [60].

There is evidence that PAR2 responses are strongly correlated with intracellular signalling mediated by the TF cytoplasmic domain [61]. Indeed, PAR2 activation mediates TF cytoplasmic domain phosphorylation. In a murine model of spontaneous breast cancer development, deletion of the TF cytoplasmic domain induced the persistence of adenoma and the delayed development of invasive carcinoma that is dependent on the angiogenic switch. A similar phenotype was also observed in PAR2^−/−^ mice, as well as in double-deficient mice, further linking TF and PAR2 signalling in the spontaneous development of invasive breast cancer [62]. Consistent with these findings, human breast cancer specimens showed marked overexpression of both PAR2 and TF antigens in invasive tumour cells [63].

In addition to PAR2, several pro-tumoural responses might be evoked by PAR1 [58,64]. Remarkably, microarray studies employing tumour cell lines strongly suggest that PAR1 and PAR2 activation induce overlapping pro-tumoural responses [59]. PAR1 has been recognized as an oncogene, promoting transformation in NIH 3T3 cells [65]. In addition, enforced expression of PAR1 promotes *in vivo* tumour growth in the human breast cancer cell line MCF-7 [66]. Yin et al. [67] have demonstrated that PAR1 mediates angiogenesis through VEGF production in carcinoma and melanoma models. Interestingly, it has been proposed that MMP-1 might be a relevant PAR1 activator in the tumour microenvironment [66,68].

Altogether, these lines of evidence indicate that the presence of TF and blood coagulation enzymes in the tumour microenvironment plays an important role in neoplastic progression, particularly through activation of PAR1 and PAR2 receptors.

## INTRAVASCULAR ACTIVATION OF BLOOD COAGULATION IN CANCER

### Molecular mechanisms

Under specific conditions, TF can be detected in the plasma circulation at abnormally elevated concentrations, where it is found mainly incorporated into tumour-derived MVs. This condition allows for the formation of the TF/FVIIa complex and subsequent intravascular activation of blood coagulation reactions, which are correlated with thrombosis occurrence. MVs are regarded as vesicular structures that are generated from the outward blebbing of the plasma membrane of various cell types, including normal and malignant cells [69]. This phenomenon is closely associated with PS exposure [70]. These MVs present with heterogeneous size (with a diameter ranging from 0.1 to 1 μm) and composition, which comprise various cell surface proteins and lipids, as well as cytoplasmic molecules, such as nucleic acids and proteins. MVs have long been studied in the context of the coagulation system [71,72]. More recently, different reports have shown that MVs participate in different aspects of tumour biology, including the activation of blood coagulation *in vitro* and *in vivo* [17,73], angiogenesis and metastasis [74,75].

Various studies have demonstrated the presence of TF on the surface of tumour MVs, likely supporting the formation of the TF/FVIIa complex [17,75–77]. In addition, PS exposure on tumour MVs promotes the assembly of the procoagulant prothrombinase complex [27,78], contributing along with TF to the propagation of the coagulation cascade. In parallel, most vascular cells exposed to procoagulant, proinflammatory or apoptotic stimuli can shed PS-exposing MVs, which may carry other components from their original cells, such as TF. Because of their PS content, MVs derived from activated platelets, for example, allow for the assembly of procoagulant complexes dependent on negatively charged membranes, contributing to thrombin generation [14,71]. TF-bearing MVs originating from activated monocytes [79] can also function as suitable surfaces for FVIIa binding. Moreover, these MVs can bind to sites of vascular injury through the interaction of P-selectin, present in activated platelets, and its ligand PSGL-1 (P-selectin glycoprotein ligand 1) [80,81]. Subsequently, they may incorporate and transfer TF and other proteins to the membrane of PS-exposing activated platelets, creating a more effective procoagulant surface and enhancing thrombin formation [82,83].

**Figure 2:**
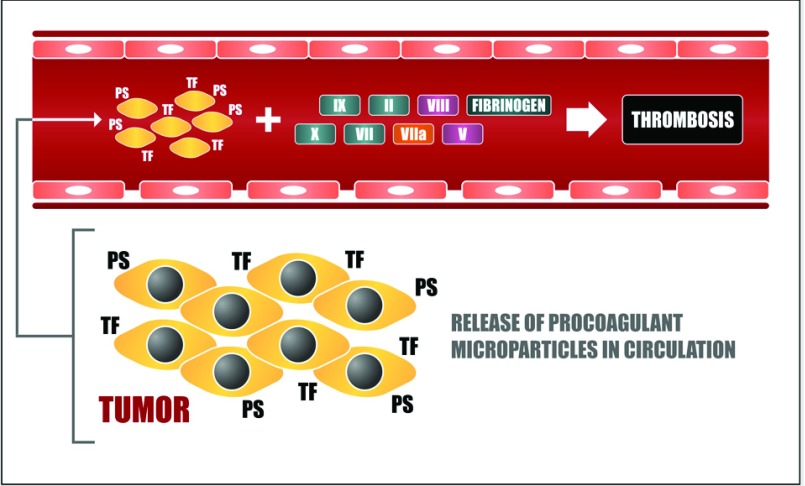
Intravascular activation of blood coagulation in cancer Tumour cells may secrete TF-bearing MVs that also expose PS on their surface. Procoagulant MVs released by tumour cells can reach the plasma circulation and activate blood coagulation reactions thus contributing to thrombus formation in the intravascular environment.

Several studies suggest that circulating MVs are key players in cancer-associated thrombosis (Figure 2). The presence of MVs in plasma has been used to explain why cancer patients may present a thromboembolic event distant from the location of tumour development. Indeed, previous studies have demonstrated that TF antigen was readily detected in the circulation of mice bearing orthotopically grown human pancreatic cancer [84,85] and murine melanoma [17]. Studies employing samples of cancer patients or plasma samples collected from tumour-bearing mice have also shown that circulating MVs may originate from platelets, monocytes and endothelium, but mostly from the cancer cells themselves [17,81,84–87]. Finally, the presence of elevated levels of TF-positive MVs in plasma has been related to the thrombogenic activity of tumour cell lines *in vivo* [81,84,85], as well as the thrombophilic state observed in cancer patients of different origins [86–89]. Other lines of clinical evidence corroborate the *in vivo* procoagulant activity of MVs, such as the strong correlation between elevated numbers of TF-positive, rather than PS-positive, MVs in human plasma and the increased risk of developing thromboembolic complications, as observed in various studies [90–93]. Conversely, in rare severe syndromes, a greater tendency for haemorrhagic events and the reduced levels of MVs have also been associated [94,95].

In addition to procoagulant MVs, other mechanisms have been proposed to explain the occurrence of cancer-associated thrombotic events. Mucins are highly glycosylated proteins that become aberrantly glycosylated in carcinomas and then are inappropriately secreted into the bloodstream. Carcinoma mucins have long been implicated in thrombus occurrence *in vivo* [96,97]. Mucins often display glycans that mediate pathological interaction with the selectin family of adhesion molecules [98]. Selectin–mucin interactions have been implicated in the haematogenous phase of tumour metastasis [99]. More recently, it was demonstrated that carcinoma mucins promote the formation of platelet-rich microthrombi *in vivo*, through adhesion-dependent, bidirectional signalling in neutrophils and platelets [98,100].

In a murine model of chronic myelogenous leukaemia, Demers et al. observed that cancer predisposes the release of chromatin into blood through the generation of NETs (neutrophil extracellular traps). Furthermore, mammary and lung carcinoma tumour-bearing mice showed an increase in peripheral blood neutrophils sensitized toward NET formation, as well as spontaneous thrombosis associated with NET generation [101].

### Impact of blood coagulation activation on tumour dissemination

As mentioned previously, studies employing cultured cells as well as patient specimens have shown that a strong correlation exists between TF expression and aggressive tumour behaviour [102,103]. TF expression correlates with increased tumour angiogenesis, as reported in studies on patient samples from non-small cell lung, colorectal, hepatocellular and pancreatic cancer [35,104–106]. It is therefore proposed that TF expression leads to an unbalanced production of anti- and/or proangiogenic factors such as VEGF that favours increased tumour vasculature [107,108] and certainly affects the metastatic process.

In particular, TF procoagulant activity, which is mediated by its extracellular domain, has been correlated with the metastatic potential of tumour cells. This activity is essential for the establishment of cancer cells in sites distal to the primary tumour, as demonstrated in melanoma, breast cancer and fibrosarcoma models [16,57,109]. Consistent with these findings, genetically altered mice defective in different coagulation proteins, such as prothrombin, fibrinogen and factor XIII, show decreased susceptibility to experimental metastasis [109–111]. In addition, Langer et al. reported that haemophilia A mice, which lack FVIII, are significantly protected against the metastatic potential of murine melanoma cells [112]. Overall, these studies support a role for blood-clotting proteins in tumour metastasis. In the initial phases of the metastatic process, fibrin deposition and platelet recruitment by thrombin in the intravascular environment seem to be crucial to the survival of tumour cells that are adhered to the endothelial layer. These events protect malignant cell elimination by NK (natural killer) cells, allowing their stable adhesion and posterior dissemination [39,58,113,114]. Gil-Bernabé et al. also proposed a mechanism in which TF-dependent clot formation recruits macrophages that are essential for *in vivo* tumour cell survival, suggesting a crucial role for coagulation in the establishment of a premetastatic niche [115]. This effect was independent of NK cells.

Activation of PAR1 by thrombin on tumour cells has also been linked to the facilitation of tumour metastasis [116]. Notably, Bromberg et al. [117] showed that the overexpression of PAR1 in low TF-expressing tumour cells is not sufficient to increase their metastatic potential. However, the acquisition of the pro-metastatic role of PAR1 *in vivo* is observed upon the co-expression of PAR1 and TF on tumour cells. In fact, several adhesion molecules, cytokines, growth factors and proteases have been identified as downstream targets of PAR1 and have been shown to modulate tumour cell metastasis [64].

In contrast to PAR1 inhibition, blocking PAR2 signalling in tumour cells has a minor impact on tumour metastasis. In this context, a non-anticoagulant monoclonal antibody that blocks TF-mediated signalling through PAR2 exhibited no effect on tumour cell dissemination in a breast cancer model [57]. On the other hand, *in vitro* assays employing cell lines demonstrated that PAR2 enhances migratory and invasive properties, which may be relevant in terms of the acquisition of a pro-metastatic phenotype *in vivo* [118,119].

In addition to its procoagulant role, the exposure of PS on cellular membranes and membrane-derived MVs can stimulate several anti-inflammatory responses that are involved in malignant processes. In this context, our group demonstrated that tumour-derived MVs also favour the establishment of melanoma metastasis in a PS-dependent manner, possibly by down-regulating the host's inflammatory and/or anti-tumoural immune responses [75]. Nevertheless, it is important to note that a pro-inflammatory cargo of MVs has also been shown [69].

## THERAPIES TARGETING BLOOD COAGULATION-RELATED PROTEINS

Given the proposed role of blood coagulation proteins in tumour progression, it has been hypothesized that targeting specific blood clotting proteins or PARs could serve as an adjuvant therapy in cancer. It has been well appreciated that antibodies that block the procoagulant function of TF attenuate metastasis [16]. Furthermore, the role of coagulation activation in tumour cell dissemination is supported by the antimetastatic effect of several anticoagulants, including the thrombin inhibitor hirudin [113,120]; and FXa inhibitors, such as NAP5 (Nematode Anticoagulant Protein 5) [121] and ACAP (*Ancylostoma caninum* anticoagulant peptide) [122]. It is noteworthy that most of these studies applied experimental models of metastasis, involving the direct injection of tumour cells into the circulation. In addition, the administration of anticoagulant agents concomitantly to or shortly after tumour cells inoculation was shown to be required for the ability of these molecules to inhibit metastasis. Altogether, these observations indicate that the inhibition of coagulation may affect the early steps of tumour spreading after intravasation, including the escape of immune responses, arrest at microvascular beds, and extravasation to a new organ. However, the effect of these anticoagulants on the initial steps of tumour dissemination, such as tumour cell detachment from the extracellular matrix, invasion of surrounding tissues and access to the systemic circulation, cannot be disregarded, and could be further examined in spontaneous metastasis models.

Because TF is suggested to play an important role in tumour biology, it has been postulated that the specific TF inhibitors attenuate cancer progression. Our group has extensively worked with ixolaris, a tick 140-amino acid salivary protein containing ten cysteines and two Kunitz-like domains. Ixolaris binds to FXa or FX as scaffolds for the inhibition of the TF/FVIIa complex, in which the FVIIa catalytic site is inactivated, as previously demonstrated by the inhibition of macromolecular (i.e., FX and FIX) substrates [123]. Ixolaris does not bind to the active site cleft of FXa. Instead, complex formation is mediated by the FXa heparin-binding exosite [124]. In addition, ixolaris interacts with circulating zymogen FX through a precursor state of the heparin-binding exosite [125], explaining its potent and long-lasting antithrombotic activity [126]. We have demonstrated that ixolaris blocks the primary growth of human glioblastoma (U87-MG) and murine melanoma (B16F10) cells in animal models, and this effect is accompanied by a significant decrease in VEGF expression as well as diminished tumour angiogenesis [127,128]. We further demonstrated that ixolaris blocks PAR2-mediated signalling in human breast tumour cells, thus accounting for the reduced production of pro-tumoural molecules [129]. NAPc2 is a TF/FVIIa inhibitor characterized from the haematophagous nematode *Ancylostoma caninum* [130]. It displays an anticoagulant mechanism of action that is similar to ixolaris and it inhibits primary tumour growth and metastasis in animal models [131]. NAPc2 has been recently evaluated in phase 1/phase 2 trial, which analysed tumour progression and metastases in metastatic colon cancer [132]. Also, a small FVIIa inhibitor, PCI-27483, is currently under a phase 2 study in advanced pancreatic cancer patients [133]. Finally, we have demonstrated that lufaxin, a specific FXa inhibitor, blocks PAR2 signalling in tumour cells [134], demonstrating that this approach may be further evaluated as the antitumour mechanism of other molecules.

It must be emphasized that a great challenge in proposing the use of anticoagulant molecules as adjuvants for the treatment of cancer is the increased tendency of patients with cancer for bleeding complications [135,136]. In this context, agents that could dissociate coagulation and signalling-related responses in tumour cells would be of great interest in this field.

## CONCLUSION AND PERSPECTIVES

A substantial amount of data implicates TF in cancer-associated hypercoagulable states. In addition, TF-dependent activation of coagulation proteases and PARs seem to be crucial for tumour growth, angiogenesis and metastasis. Thus, therapeutic strategies targeting some effector molecules of blood coagulation activation could attenuate their deleterious effects in cancer, certainly affecting the morbidity and survival of cancer patients. Nevertheless, additional studies are needed to identify mechanistic insights into the role of blood coagulation activation and coagulation-dependent signalling pathways in cancer biology, as well as recognize potential blood coagulation targets to inhibit cancer progression without compromising haemostasis.

## References

[1] Trousseau A. (1865). Phlegmasia alba dolens. Clin. Med. Hotel-Dieu Paris.

[2] Rickles F. R., Levine M. N. (2001). Epidemiology of thrombosis in cancer. Acta Haematol..

[3] Mandala M., Ferretti G., Cremonesi M., Cazzaniga M., Curigliano G., Barni S. (2003). Venous thromboembolism and cancer: new issues for an old topic. Crit. Rev. Oncol. Hematol..

[4] Hoffman R., Haim N., Brenner B. (2001). Cancer and thrombosis revisited. Blood Rev..

[5] Donati M. B. (1995). Cancer and thrombosis: from *Phlegmasia alba dolens* to transgenic mice. Thromb. Haemost..

[6] Khorana A. A., Francis C. W., Culakova E., Kuderer N. M., Lyman G. H. (2007). Thromboembolism is a leading cause of death in cancer patients receiving outpatient chemotherapy. J. Thromb. Haemost..

[7] Rickles F. R., Edwards R. L. (1983). Activation of blood coagulation in cancer: Trousseau's syndrome revisited. Blood.

[8] Sorensen H. T., Mellemkjaer L., Olsen J. H., Baron J. A. (2000). Prognosis of cancers associated with venous thromboembolism. N. Engl. J. Med..

[9] Levitan N., Dowlati A., Remick S. C., Tahsildar H. I., Sivinski L. D., Beyth R., Rimm A. A. (1999). Rates of initial and recurrent thromboembolic disease among patients with malignancy versus those without malignancy. Risk analysis using Medicare claims data. Medicine (Baltimore).

[10] Sallah S., Wan J. Y., Nguyen N. P., Hanrahan L. R., Sigounas G. (2001). Disseminated intravascular coagulation in solid tumors: clinical and pathologic study. Thromb. Haemost..

[11] Degen J. L., Palumbo J. S. (2012). Hemostatic factors, innate immunity and malignancy. Thromb. Res..

[12] van den Berg Y. W., Osanto S., Reitsma P. H., Versteeg H. H. (2012). The relationship between tissue factor and cancer progression: insights from bench and bedside. Blood.

[13] Francis J. L., Biggerstaff J., Amirkhosravi A. (1998). Hemostasis and malignancy. Sem. Thromb. Hemost..

[14] Zwicker J. I., Furie B. C., Furie B. (2007). Cancer-associated thrombosis. Crit. Rev. Oncol. Hematol..

[15] Ruf W., Edgington T. S. (1994). Structural biology of tissue factor, the initiator of thrombogenesis *in vivo*. FASEB J..

[16] Mueller B. M., Reisfeld R. A., Edgington T. S., Ruf W. (1992). Expression of tissue factor by melanoma cells promotes efficient hematogenous metastasis. Proc. Natl. Acad. Sci. U.S.A..

[17] Lima L. G., Oliveira A. S., Campos L. C., Bonamino M., Chammas R., Werneck C., Vicente C. P., Barcinski M. A., Petersen L. C., Monteiro R. Q. (2011). Malignant transformation in melanocytes is associated with increased production of procoagulant microvesicles. Thromb. Haemost..

[18] Gerotziafas G. T., Galea V., Mbemba E., Khaterchi A., Sassi M., Baccouche H., Prengel C., van Dreden P., Hatmi M. (2012). Tissue factor over-expression by human pancreatic cancer cells BXPC3 is related to higher prothrombotic potential as compared to breast cancer cells MCF7. Thromb. Res..

[19] Rak J., Milsom C., Magnus N., Yu J. (2009). Tissue factor in tumour progression. Best Pract. Res. Clin. Haematol..

[20] Yu J. L., May L., Lhotak V., Shahrzad S., Shirasawa S., Weitz J. I., Coomber B. L., Mackman N., Rak J. W. (2005). Oncogenic events regulate tissue factor expression in colorectal cancer cells: implications for tumor progression and angiogenesis. Blood.

[21] Milsom C. C., Yu J. L., Mackman N., Micallef J., Anderson G. M., Guha A., Rak J. W. (2008). Tissue factor regulation by epidermal growth factor receptor and epithelial-to-mesenchymal transitions: effect on tumor initiation and angiogenesis. Cancer Res..

[22] Rong Y., Post D. E., Pieper R. O., Durden D. L., van Meir E. G., Brat D. J. (2005). PTEN and hypoxia regulate tissue factor expression and plasma coagulation by glioblastoma. Cancer Res..

[23] Zhang X., Yu H., Lou J. R., Zheng J., Zhu H., Popescu N., Lupu F., Lind S. E., Ding W. (2011). MicroRNA-19 (miR-19) regulates tissue factor expression in breast cancer cells. J. Biol. Chem..

[24] Utsugi T., Schroit A. J., Connor J., Bucana C. D., Fidler I. J. (1991). Elevated expression of phosphatidylserine in the outer membrane leaflet of human tumor cells and recognition by activated human blood monocytes. Cancer Res..

[25] Tormoen G. W., Cianchetti F. A., Bock P. E., McCarty O. J. T. (2012). Development of coagulation factor probes for the identification of procoagulant circulating tumor cells. Front. Oncol..

[26] Tormoen G. W., Haley K. M., Levine R. L., McCarty O. J. T. (2012). Do circulating tumor cells play a role in coagulation and thrombosis?. Front. Oncol..

[27] VanDeWater L., Tracy P. B., Aronson D., Mann K. G., Dvorak H. F. (1985). Tumor cell generation of thrombin via functional prothrombinase assembly. Cancer Res..

[28] Rickles F. R., Falanga A. (2001). Molecular basis for the relationship between thrombosis and cancer. Thromb. Res..

[29] Kirszberg C., Rumjanek V. M., Monteiro R. Q. (2005). Assembly and regulation of prothrombinase complex on B16F10 melanoma cells. Thromb. Res..

[30] Kirszberg C., Lima L. G., Da Silva de O. A., Pickering W., Gray E., Barrowcliffe T. W., Rumjanek V. M., Monteiro R. Q. (2009). Simultaneous tissue factor expression and phosphatidylserine exposure account for the highly procoagulant pattern of melanoma cell lines. Melanoma Res..

[31] Fernandes R. S., Kirszberg C., Rumjanek V. M., Monteiro R. Q. (2006). On the molecular mechanisms for the highly procoagulant pattern of C6 glioma cells. J. Thromb. Haemost..

[32] Zacharski L. R., Dunwiddie C., Nutt E. M., Hunt J., Memoli V. A. (1991). Cellular localization of activated factor X by Xa-specific probes. Thromb. Haemost..

[33] Zacharski L. R., Memoli V. A., Morain W. D., Schlaeppi J. M., Rousseau S. M. (1995). Cellular localization of enzymatically active thrombin in intact human tissues by hirudin binding. Thromb. Haemost..

[34] Buller H. R., van Doormaal F. F., van Sluis G. L., Kamphuisen P. W. (2007). Cancer and thrombosis: from molecular mechanisms to clinical presentations. J. Thromb. Haemost..

[35] Khorana A. A., Ahrendt S. A., Ryan C. K., Francis C. W., Hruban R. H., Hu Y. C., Hostetter G., Harvey J., Taubman M. B. (2007). Tissue factor expression, angiogenesis, and thrombosis in pancreatic cancer. Clin. Cancer Res..

[36] Abe K., Shoji M., Chen J., Bierhaus A., Danave I., Micko C., Casper K., Dillehay D. L., Nawroth P. P., Rickles F. R. (1999). Regulation of vascular endothelial growth factor production and angiogenesis by the cytoplasmic tail of tissue factor. Proc. Natl. Acad. Sci. U.S.A..

[37] Coughlin S. R. (2005). Protease-activated receptors in hemostasis, thrombosis and vascular biology. J. Thromb. Haemost..

[38] Riewald M., Ruf W. (2001). Mechanistic coupling of protease signaling and initiation of coagulation by tissue factor. Proc. Natl. Acad. Sci. U.S.A..

[39] Camerer E., Qazi A. A., Duong D. N., Cornelissen I., Advincula R., Coughlin S. R. (2004). Platelets, protease-activated receptors, and fibrinogen in hematogenous metastasis. Blood.

[40] Ossovskaya V. S., Bunnett N. W. (2004). Protease-activated receptors: contribution to physiology and disease. Physiol. Rev..

[41] Henrikson K. P., Salazar S. L., Fenton J. W., Pentecost B. T. (1999). Role of thrombin receptor in breast cancer invasiveness. Br. J. Cancer.

[42] Ikeda O., Egami H., Ishiko T., Ishikawa S., Kamohara H., Hidaka H., Mita S., Ogawa M. (2003). Expression of proteinase-activated receptor-2 in human pancreatic cancer: a possible relation to cancer invasion and induction of fibrosis. Int. J. Oncol..

[43] Yin Y. J., Salah Z., Grisaru-Granovsky S., Cohen I., Even-Ram S. C., Maoz M., Uziely B., Peretz T., Bar-Shavit R. (2003). Human protease-activated receptor 1 expression in malignant epithelia: a role in invasiveness. Arterioscler. Thromb. Vasc. Biol..

[44] Ribeiro F. S., Simao T. A., Amoedo N. D., Andreollo N. A., Lopes L. R., Acatauassu R., Rumjanek F. D., Albano R. M., Pinto L. F., Monteiro R. Q. (2009). Evidence for increased expression of tissue factor and protease-activated receptor-1 in human esophageal cancer. Oncol. Rep..

[45] Veiga C. S., Carneiro-Lobo T. C., Coelho C. J., Carvalho S. M., Maia R. C., Vasconcelos F. C., Abdelhay E., Mencalha A. L., Ferreira A. F., Castro F. A., Monteiro R. Q. (2011). Increased expression of protease-activated receptor 1 (PAR-1) in human leukemias. Blood Cells Mol. Dis..

[46] Schaffner F., Ruf W. (2008). Tissue factor and protease-activated receptor signaling in cancer. Semin. Thromb. Hemost..

[47] Coughlin S. R. (2000). Thrombin signalling and protease-activated receptors. Nature.

[48] Maragoudakis M. E., Tsopanoglou N. E., Andriopoulou P., Maragoudakis M. M. (2000). Effects of thrombin/thrombosis in angiogenesis and tumour progression. Matrix Biol..

[49] Nierodzik M. L., Karpatkin S. (2006). Thrombin induces tumor growth, metastasis, and angiogenesis: evidence for a thrombin-regulated dormant tumor phenotype. Cancer Cell.

[50] Chen J., Bierhaus A., Schiekofer S., Andrassy M., Chen B., Stern D. M., Nawroth P. P. (2001). Tissue factor–a receptor involved in the control of cellular properties, including angiogenesis. Thromb. Haemost..

[51] Riewald M., Ruf W. (2002). Orchestration of coagulation protease signaling by tissue factor. Trends Cardiovasc. Med..

[52] Ruf W., Dorfleutner A., Riewald M. (2003). Specificity of coagulation factor signaling. J. Thromb. Haemost..

[53] Jiang X., Bailly M. A., Panetti T. S., Cappello M., Konigsberg W. H., Bromberg M. E. (2004). Formation of tissue factor-factor VIIa-factor Xa complex promotes cellular signaling and migration of human breast cancer cells. J. Thromb. Haemost..

[54] Belting M., Dorrell M. I., Sandgren S., Aguilar E., Ahamed J., Dorfleutner A., Carmeliet P., Mueller B. M., Friedlander M., Ruf W. (2004). Regulation of angiogenesis by tissue factor cytoplasmic domain signaling. Nat. Med..

[55] Hjortoe G. M., Petersen L. C., Albrektsen T., Sorensen B. B., Norby P. L., Mandal S. K., Pendurthi U. R., Rao L. V. (2004). Tissue factor-factor VIIa-specific up-regulation of IL-8 expression in MDA-MB-231 cells is mediated by PAR-2 and results in increased cell migration. Blood.

[56] Dutra-Oliveira A., Monteiro R. Q., Mariano-Oliveira A. (2012). Protease-activated receptor-2 (PAR2) mediates VEGF production through the ERK1/2 pathway in human glioblastoma cell lines. Biochem. Biophys. Res. Commun..

[57] Versteeg H. H., Schaffner F., Kerver M., Petersen H. H., Ahamed J., Felding-Habermann B., Takada Y., Mueller B. M., Ruf W. (2008). Inhibition of tissue factor signaling suppresses tumor growth. Blood.

[58] Ruf W., Disse J., Carneiro-Lobo T. C., Yokota N., Schaffner F. (2011). Tissue factor and cell signalling in cancer progression and thrombosis. J. Thromb. Haemost..

[59] Albrektsen T., Sorensen B. B., Hjortoe G. M., Fleckner J., Rao L. V., Petersen L. C. (2007). Transcriptional program induced by factor VIIa-tissue factor, PAR1 and PAR2 in MDA-MB-231 cells. J. Thromb. Haemost..

[60] Versteeg H. H., Schaffner F., Kerver M., Ellies L. G., Andrade-Gordon P., Mueller B. M., Ruf W. (2008). Protease-activated receptor (PAR) 2, but not PAR1, signaling promotes the development of mammary adenocarcinoma in polyoma middle T mice. Cancer Res..

[61] Ahamed J., Ruf W. (2004). Protease-activated receptor 2-dependent phosphorylation of the tissue factor cytoplasmic domain. J. Biol. Chem..

[62] Schaffner F., Versteeg H. H., Schillert A., Yokota N., Petersen L. C., Mueller B. M., Ruf W. (2010). Cooperation of tissue factor cytoplasmic domain and PAR2 signaling in breast cancer development. Blood.

[63] Ryden L., Grabau D., Schaffner F., Jonsson P. E., Ruf W., Belting M. (2010). Evidence for tissue factor phosphorylation and its correlation with protease activated receptor expression and the prognosis of primary breast cancer. Int. J. Cancer.

[64] Villares G. J., Zigler M., Bar-Eli M. (2011). The emerging role of the thrombin receptor (PAR-1) in melanoma metastasis-a possible therapeutic target. Oncotarget.

[65] Whitehead I., Kirk H., Kay R. (1995). Expression cloning of oncogenes by retroviral transfer of cDNA libraries. Mol. Cell. Biol..

[66] Boire A., Covic L., Agarwal A., Jacques S., Sherifi S., Kuliopulos A. (2005). PAR1 is a matrix metalloprotease-1 receptor that promotes invasion and tumorigenesis of breast cancer cells. Cell.

[67] Yin Y. J., Salah Z., Maoz M., Even-Ram S. C., Ochayon S., Neufeld G., Katzav S., Bar-Shavit R. (2003). Oncogenic transformation induces tumor angiogenesis: a role for PAR1 activation. FASEB J..

[68] Kim S. J., Shin J. Y., Lee K. D., Bae Y. K., Choi I. J., Park S. H., Chun K. H. (2011). Galectin-3 facilitates cell motility in gastric cancer by up-regulating protease-activated receptor-1 (PAR-1) and matrix metalloproteinase-1 (MMP-1). PLoS ONE.

[69] Lee T. H., D’Asti E., Magnus N., Al-Nedawi K., Meehan B., Rak J. (2011). Microvesicles as mediators of intercellular communication in cancer-the emerging science of cellular ‘debris’. Semin. Immunopathol..

[70] Zwaal R. F., Schroit A. J. (1997). Pathophysiologic implications of membrane phospholipid asymmetry in blood cells. Blood.

[71] Diamant M., Tushuizen M. E., Sturk A., Nieuwland R. (2004). Cellular microparticles: new players in the field of vascular disease?. Eur. J. Clin. Invest..

[72] Aharon A., Brenner B. (2009). Microparticles, thrombosis and cancer. Best Pract. Res. Clin. Haematol..

[73] Dvorak H. F., Quay S. C., Orenstein N. S., Dvorak A. M., Hahn P., Bitzer A. M., Carvalho A. C. (1981). Tumor shedding and coagulation. Science.

[74] Janowska-Wieczorek A., Wysoczynski M., Kijowski J., Marquez-Curtis L., Machalinski B., Ratajczak J., Ratajczak M. Z. (2005). Microvesicles derived from activated platelets induce metastasis and angiogenesis in lung cancer. Int. J. Cancer.

[75] Lima L. G., Chammas R., Monteiro R. Q., Moreira M. E., Barcinski M. A. (2009). Tumor-derived microvesicles modulate the establishment of metastatic melanoma in a phosphatidylserine-dependent manner. Cancer Lett..

[76] Bastida E., Ordinas A., Escolar G., Jamieson G. A. (1984). Tissue factor in microvesicles shed from U87MG human glioblastoma cells induces coagulation, platelet aggregation, and thrombogenesis. Blood.

[77] Yu J. L., Rak J. W. (2004). Shedding of tissue factor (TF)-containing microparticles rather than alternatively spliced TF is the main source of TF activity released from human cancer cells. J. Thromb. Haemost..

[78] Dvorak H. F., Van D. L., Bitzer A. M., Dvorak A. M., Anderson D., Harvey V. S., Bach R., Davis G. L., DeWolf W., Carvalho A. C. (1983). Procoagulant activity associated with plasma membrane vesicles shed by cultured tumor cells. Cancer Res..

[79] Satta N., Toti F., Feugeas O., Bohbot A., Dachary-Prigent J., Eschwege V., Hedman H., Freyssinet J. M. (1994). Monocyte vesiculation is a possible mechanism for dissemination of membrane-associated procoagulant activities and adhesion molecules after stimulation by lipopolysaccharide. J. Immunol..

[80] McGregor L., Martin J., McGregor J. L. (2006). Platelet-leukocyte aggregates and derived microparticles in inflammation, vascular remodelling and thrombosis. Front. Biosci..

[81] Thomas G. M., Panicot-Dubois L., Lacroix R., Dignat-George F., Lombardo D., Dubois C. (2009). Cancer cell-derived microparticles bearing P-selectin glycoprotein ligand 1 accelerate thrombus formation *in vivo*. J. Exp. Med..

[82] Rauch U., Bonderman D., Bohrmann B., Badimon J. J., Himber J., Riederer M. A., Nemerson Y. (2000). Transfer of tissue factor from leukocytes to platelets is mediated by CD15 and tissue factor. Blood.

[83] Eilertsen K. E., Osterud B. (2004). Tissue factor: (patho)physiology and cellular biology. Blood Coagul. Fibrinolysis.

[84] Davila M., Amirkhosravi A., Coll E., Desai H., Robles L., Colon J., Baker C. H., Francis J. L. (2008). Tissue factor-bearing microparticles derived from tumor cells: impact on coagulation activation. J. Thromb. Haemost..

[85] Wang J. G., Geddings J. E., Aleman M. M., Cardenas J. C., Chantrathammachart P., Williams J. C., Kirchhofer D., Bogdanov V. Y., Bach R. R., Rak J. (2012). Tumor-derived tissue factor activates coagulation and enhances thrombosis in a mouse xenograft model of human pancreatic cancer. Blood.

[86] Hron G., Kollars M., Weber H., Sagaster V., Quehenberger P., Eichinger S., Kyrle P. A., Weltermann A. (2007). Tissue factor-positive microparticles: cellular origin and association with coagulation activation in patients with colorectal cancer. Thromb. Haemost..

[87] Tesselaar M. E., Romijn F. P., van der Linden I. K., Prins F. A., Bertina R. M., Osanto S. (2007). Microparticle-associated tissue factor activity: a link between cancer and thrombosis?. J. Thromb. Haemost..

[88] Langer F., Spath B., Haubold K., Holstein K., Marx G., Wierecky J., Brummendorf T. H., Dierlamm J., Bokemeyer C., Eifrig B. (2008). Tissue factor procoagulant activity of plasma microparticles in patients with cancer-associated disseminated intravascular coagulation. Ann. Hematol..

[89] Zwicker J. I., Liebman H. A., Neuberg D., Lacroix R., Bauer K. A., Furie B. C., Furie B. (2009). Tumor-derived tissue factor-bearing microparticles are associated with venous thromboembolic events in malignancy. Clin. Cancer Res..

[90] Khan I., Zucker-Franklin D., Karpatkin S. (1975). Microthrombocytosis and platelet fragmentation associated with idiopathic/autoimmune thrombocytopenic purpura. Br. J. Haematol..

[91] Mallat Z., Benamer H., Hugel B., Benessiano J., Steg P. G., Freyssinet J. M., Tedgui A. (2000). Elevated levels of shed membrane microparticles with procoagulant potential in the peripheral circulating blood of patients with acute coronary syndromes. Circulation.

[92] Ando M., Iwata A., Ozeki Y., Tsuchiya K., Akiba T., Nihei H. (2002). Circulating platelet-derived microparticles with procoagulant activity may be a potential cause of thrombosis in uremic patients. Kidney Int..

[93] Rautou P. E., Mackman N. (2013). Microvesicles as risk markers for venous thrombosis. Expert Rev. Hematol..

[94] Gemmell C. H., Sefton M. V., Yeo E. L. (1993). Platelet-derived microparticle formation involves glycoprotein IIb–IIIa. Inhibition by RGDS and a Glanzmann's thrombasthenia defect. J. Biol. Chem..

[95] Castaman G., Yu-Feng L., Battistin E., Rodeghiero F. (1997). Characterization of a novel bleeding disorder with isolated prolonged bleeding time and deficiency of platelet microvesicle generation. Br. J. Haematol..

[96] Min K. W., Gyorkey F., Sato C. (1980). Mucin-producing adenocarcinomas and nonbacterial thrombotic endocarditis: pathogenetic role of tumor mucin. Cancer.

[97] Varki A. (2007). Trousseau's syndrome: multiple definitions and multiple mechanisms. Blood.

[98] Wahrenbrock M., Borsig L., Le D., Varki N., Varki A. (2003). Selectin-mucin interactions as a probable molecular explanation for the association of Trousseau syndrome with mucinous adenocarcinomas. J. Clin. Invest..

[99] Borsig L., Wong R., Feramisco J., Nadeau D. R., Varki N. M., Varki A. (2001). Heparin and cancer revisited: mechanistic connections involving platelets, P-selectin, carcinoma mucins, and tumor metastasis. Proc. Natl. Acad. Sci. U.S.A..

[100] Shao B., Wahrenbrock M. G., Yao L., David T., Coughlin S. R., Xia L., Varki A., McEver R. P. (2011). Carcinoma mucins trigger reciprocal activation of platelets and neutrophils in a murine model of Trousseau syndrome. Blood.

[101] Demers M., Krause D. S., Schatzberg D., Martinod K., Voorhees J. R., Fuchs T. A., Scadden D. T., Wagner D. D. (2012). Cancers predispose neutrophils to release extracellular DNA traps that contribute to cancer-associated thrombosis. Proc. Natl. Acad. Sci. U.S.A..

[102] Nitori N., Ino Y., Nakanishi Y., Yamada T., Honda K., Yanagihara K., Kosuge T., Kanai Y., Kitajima M., Hirohashi S. (2005). Prognostic significance of tissue factor in pancreatic ductal adenocarcinoma. Clin. Cancer Res..

[103] Patry G., Hovington H., Larue H., Harel F., Fradet Y., Lacombe L. (2008). Tissue factor expression correlates with disease-specific survival in patients with node-negative muscle-invasive bladder cancer. Int. J. Cancer.

[104] Sawada M., Miyake S., Ohdama S., Matsubara O., Masuda S., Yakumaru K., Yoshizawa Y. (1999). Expression of tissue factor in non-small-cell lung cancers and its relationship to metastasis. Br. J. Cancer.

[105] Nakasaki T., Wada H., Shigemori C., Miki C., Gabazza E. C., Nobori T., Nakamura S., Shiku H. (2002). Expression of tissue factor and vascular endothelial growth factor is associated with angiogenesis in colorectal cancer. Am. J. Hematol..

[106] Poon R. T., Lau C. P., Ho J. W., Yu W. C., Fan S. T., Wong J. (2003). Tissue factor expression correlates with tumor angiogenesis and invasiveness in human hepatocellular carcinoma. Clin. Cancer Res..

[107] Zhang Y., Deng Y., Luther T., Muller M., Ziegler R., Waldherr R., Stern D. M., Nawroth P. P. (1994). Tissue factor controls the balance of angiogenic and antiangiogenic properties of tumor cells in mice. J. Clin. Invest..

[108] Rak J., Milsom C., May L., Klement P., Yu J. (2006). Tissue factor in cancer and angiogenesis: the molecular link between genetic tumor progression, tumor neovascularization, and cancer coagulopathy. Semin. Thromb. Hemost..

[109] Palumbo J. S., Talmage K. E., Massari J. V., La Jeunesse C. M., Flick M. J., Kombrinck K. W., Hu Z., Barney K. A., Degen J. L. (2007). Tumor cell-associated tissue factor and circulating hemostatic factors cooperate to increase metastatic potential through natural killer cell-dependent and-independent mechanisms. Blood.

[110] Palumbo J. S., Degen J. L. (2007). Mechanisms linking tumor cell-associated procoagulant function to tumor metastasis. Thromb. Res..

[111] Palumbo J. S., Barney K. A., Blevins E. A., Shaw M. A., Mishra A., Flick M. J., Kombrinck K. W., Talmage K. E., Souri M., Ichinose A., Degen J. L. (2008). Factor XIII transglutaminase supports hematogenous tumor cell metastasis through a mechanism dependent on natural killer cell function. J. Thromb. Haemost..

[112] Langer F., Amirkhosravi A., Ingersoll S. B., Walker J. M., Spath B., Eifrig B., Bokemeyer C., Francis J. L. (2006). Experimental metastasis and primary tumor growth in mice with hemophilia A. J. Thromb. Haemost..

[113] Im J. H., Fu W., Wang H., Bhatia S. K., Hammer D. A., Kowalska M. A., Muschel R. J. (2004). Coagulation facilitates tumor cell spreading in the pulmonary vasculature during early metastatic colony formation. Cancer Res..

[114] Palumbo J. S., Talmage K. E., Massari J. V., La Jeunesse C. M., Flick M. J., Kombrinck K. W., Jirouskova M., Degen J. L. (2005). Platelets and fibrin(ogen) increase metastatic potential by impeding natural killer cell-mediated elimination of tumor cells. Blood.

[115] Gil-Bernabé A. M., Ferjancic S., Tlalka M., Zhao L., Allen P. D., Im J. H., Watson K., Hill S. A., Amirkhosravi A., Francis J. L. (2012). Recruitment of monocytes/macrophages by tissue factor-mediated coagulation is essential for metastatic cell survival and premetastatic niche establishment in mice. Blood.

[116] Nierodzik M. L., Chen K., Takeshita K., Li J. J., Huang Y. Q., Feng X. S., D’Andrea M. R., Andrade-Gordon P., Karpatkin S. (1998). Protease-activated receptor 1 (PAR-1) is required and rate-limiting for thrombin-enhanced experimental pulmonary metastasis. Blood.

[117] Bromberg M. E., Bailly M. A., Konigsberg W. H. (2001). Role of protease-activated receptor 1 in tumor metastasis promoted by tissue factor. Thromb. Haemost..

[118] Morris D. R., Ding Y., Ricks T. K., Gullapalli A., Wolfe B. L., Trejo J. (2006). Protease-activated receptor-2 is essential for factor VIIa and Xa-induced signaling, migration, and invasion of breast cancer cells. Cancer Res..

[119] Su S., Li Y., Luo Y., Sheng Y., Su Y., Padia R. N., Pan Z. K., Dong Z., Huang S. (2009). Proteinase-activated receptor 2 expression in breast cancer and its role in breast cancer cell migration. Oncogene.

[120] Esumi N., Fan D., Fidler I. J. (1991). Inhibition of murine melanoma experimental metastasis by recombinant desulfatohirudin, a highly specific thrombin inhibitor. Cancer Res..

[121] Hembrough T. A., Swartz G. M., Papathanassiu A., Vlasuk G. P., Rote W. E., Green S. J., Pribluda V. S. (2003). Tissue factor/factor VIIa inhibitors block angiogenesis and tumor growth through a nonhemostatic mechanism. Cancer Res..

[122] Donnelly K. M., Bromberg M. E., Milstone A., Madison McNiff J. M., Terwilliger G., Konigsberg W. H., Cappello M. (1998). *Ancylostoma caninum* anticoagulant peptide blocks metastasis *in vivo* and inhibits factor Xa binding to melanoma cells *in vitro*. Thromb. Haemost..

[123] Francischetti I. M., Valenzuela J. G., Andersen J. F., Mather T. N., Ribeiro J. M. (2002). Ixolaris, a novel recombinant tissue factor pathway inhibitor (TFPI) from the salivary gland of the tick, Ixodes scapularis: identification of factor X and factor Xa as scaffolds for the inhibition of factor VIIa/tissue factor complex. Blood.

[124] Monteiro R. Q., Rezaie A. R., Ribeiro J. M., Francischetti I. M. (2005). Ixolaris: a factor Xa heparin-binding exosite inhibitor. Biochem. J..

[125] Monteiro R. Q., Rezaie A. R., Bae J. S., Calvo E., Andersen J. F., Francischetti I. M. (2008). Ixolaris binding to factor X reveals a precursor state of factor Xa heparin-binding exosite. Protein Sci..

[126] Nazareth R. A., Tomaz L. S., Ortiz-Costa S., Atella G. C., Ribeiro J. M., Francischetti I. M., Monteiro R. Q. (2006). Antithrombotic properties of Ixolaris, a potent inhibitor of the extrinsic pathway of the coagulation cascade. Thromb. Haemost..

[127] Carneiro-Lobo T. C., Konig S., Machado D. E., Nasciutti L. E., Forni M. F., Francischetti I. M., Sogayar M. C., Monteiro R. Q. (2009). Ixolaris, a tissue factor inhibitor, blocks primary tumor growth and angiogenesis in a glioblastoma model. J. Thromb. Haemost..

[128] de Oliveira A. S., Lima L. G., Mariano-Oliveira A., Machado D. E., Nasciutti L. E., Andersen J. F., Petersen L. C., Francischetti I. M., Monteiro R. Q. (2012). Inhibition of tissue factor by ixolaris reduces primary tumor growth and experimental metastasis in a murine model of melanoma. Thromb. Res..

[129] Carneiro-Lobo T. C., Schaffner F., Disse J., Ostergaard H., Francischetti I. M., Monteiro R. Q., Ruf W. (2012). The tick-derived inhibitor Ixolaris prevents tissue factor signaling on tumor cells. J. Thromb. Haemost..

[130] Monteiro R. Q., Andersen J. F., Francischetti I. M. B. (2011). Hematophagy and inhibition of the extrinsic and intrinsic tenase complexes. Toxins and Hemostasis.

[131] Zhao J., Aguilar G., Palencia S., Newton E., Abo A. (2009). rNAPc2 inhibits colorectal cancer in mice through tissue factor. Clin. Cancer Res..

[132] National Institutes of Health (2007). Safety study of recombinant NAPc2 to prevent tumor progression and metastases in metastatic colon cancer. No. NCT00443573.

[133] National Institutes of Health (2009). Study of Safety and Tolerability of PCI-27483 in Patients With Pancreatic Cancer Patients Receiving Treatment With Gemcitabine. No. NCT01020006.

[134] Collin N., Assumpção T. C., Mizurini D. M., Gilmore D. C., Dutra-Oliveira A., Kotsyfakis M., Sá-Nunes A., Teixeira C., Ribeiro J. M., Monteiro R. Q. (2012). Lufaxin, a novel factor Xa inhibitor from the salivary gland of the sand fly Lutzomya longipalpis blocks protease-activated receptor 2 activation and inhibits inflammation and thrombosis *in vivo*. Arterioscler. Thromb. Vasc. Biol..

[135] Trujillo-Santos J., Nieto J. A., Ruíz-Gamietea A., López-Jiménez L., García-Bragado F., Quintavalla R., Monreal M., RIETE, Investigators (2010). Bleeding complications associated with anticoagulant therapy in patients with cancer. Thromb. Res..

[136] Prandoni P., Lensing A. W., Piccioli A., Bernardi E., Simioni P., Girolami B., Marchiori A., Sabbion P., Prins M. H., Noventa F., Girolami A. (2002). Recurrent venous thromboembolism and bleeding complications during anticoagulant treatment in patients with cancer and venous thrombosis. Blood.

